# Personality in Chronic Headache: A Systematic Review with Meta-Analysis

**DOI:** 10.1155/2023/6685372

**Published:** 2023-08-28

**Authors:** Sara Bottiroli, Alessia Renzi, Elena Ballante, Roberto De Icco, Grazia Sances, Annalisa Tanzilli, Tomaso Vecchi, Cristina Tassorelli, Federica Galli

**Affiliations:** ^1^Giustino Fortunato University, Benevento, Italy; ^2^Headache Science and Neurorehabilitation Centre, IRCCS C. Mondino Foundation, Pavia, Italy; ^3^Department of Dynamic and Clinical Psychology and Health Studies, “Sapienza” University of Rome, Rome, Italy; ^4^BioData Science Unit, IRCCS C. Mondino Foundation, Pavia, Italy; ^5^Department of Political and Social Sciences, University of Pavia, Italy; ^6^Department of Brain and Behavioral Sciences, University of Pavia, Italy

## Abstract

**Background:**

Chronic headache (CH) is a condition that includes different subtypes of headaches and that can impair different life domains. Personality traits can play a relevant role both in the development and in coping with this medical condition. The first aim of the present study is to realize a systematic review of the personality traits associated with CH compared to healthy controls; the second objective is to carry out a quantitative meta-analysis with the studies using the same instrument to assess personality traits.

**Method:**

The literature search encompassed articles published from 1988 until December 2022 on the major databases in the field of health and social sciences: PubMed, Scopus, PsychInfo, and Web of Science.

**Results:**

Thirteen studies were included in the systematic review, but only three studies were deeply explored in a meta-analysis since the only ones used a common instrument for personality assessment (Minnesota Multiphasic Personality Inventory). According to the meta-analysis, different subtypes of CH patients scored higher than healthy controls on Hypochondriasis and Hysteria Scales. The systematic review showed higher levels of depressive and anxious personality dimensions and pain catastrophizing in CH compared to healthy controls. Moreover, frequent-chronic forms and medication-overuse headache were the most symptomatic and frail categories showing higher levels of dysfunctional personality traits and psychopathological symptoms.

**Conclusions:**

These results seem to confirm a “neurotic profile” in patients suffering from CH. The identification of the main personality traits involved in the onset and maintenance of headache disorders represents an important objective for developing psychological interventions.

## 1. Introduction

Chronic pain (CP), defined as pain lasting more than 3 months, is a significant healthcare challenge with considerable economic costs and psychological burden. Prevalence rates of CP vary between 11% and 40% [1], with chronic headache (CH) representing a consistent group of affected people. The 3rd edition of the International Classification of Headache Disorders (ICHD) [2] identifies different subtypes of chronic headache (CH), all characterized by the presence of headache on >15 days/month. These subtypes include chronic migraine (CM), chronic tension-type headache (CTTH), and medication-overuse headache (MOH) [2]. The prevalence in the general adult population of CM is about 2%, that of CTTH ranges from 1.7% to 2.2%, and that of MOH is 1-2% [3–6].

The investigation of psychiatric comorbidity in headache disease dates back to the beginning of the 90s [7], with a focus on the role of anxiety and mood disorders in migraines. The association among migraine, anxiety, and depression is strong, both in clinical and community samples [8]. However, this comorbid association is not specific to migraine, but it has been evidenced in patients with CH as well [7]. Although estimates can differ, about 47% of population-based samples of people with migraine reported comorbid depression, and about 58% suffer from comorbid anxiety [9–11]. Both these psychopathological conditions are more represented in people with CM compared to people affected by episodic forms [9, 11, 12]. Moreover, people with episodic migraine (EM) having comorbid depression are more likely to progress to CM in the following year, thus configuring depression as a risk factor for disease synchronization [9, 13]. Suffering from both migraine and psychiatric disorders (i.e., anxiety and depression) denotes worsened symptomatology for each condition with greater health expenditures and medication use compared to migraineurs without psychopathological comorbidities, reduced quality of life (QoL), and increased burden and frailty associated with migraine [9, 14, 15]. However, the specific mechanisms and the exact direction (headache causing anxiety/depression or vice versa) of this association remain unknown. According to the biopsychosocial model of health [16, 17], it exists a complex interaction between psychological, psychosocial, and biological aspects, reciprocally influencing each other. Consequently, the expression of headache/migraine is not fixed for all sufferers, as it results from the interaction of these factors, which can negatively influence the course of the disease and enhance dysfunctional pain processing.

In this light, personality represents a relatively stable pattern of thinking, feeling, or behaving that tends to be consistent over time and across relevant situations [18]. Accordingly, personality denotes the kind of adaptation the individual shows to the external environment and the related lifestyle patterns [19]. Over time, many theories on personality structure have been proposed [20], and in both the clinical and reasearch area, the most widely employed are (a) the psychobiological model [21], considering seven dimensions that are novelty seeking, harm avoidance, reward dependence and persistence, self-directedness, cooperativeness, and self-transcendence, (b) the Big Five Model [22], exploring five main dimensions that are extraversion, agreeableness, conscientiousness, negative emotionality, and openness to experience, and (c) the Eysenck's three-factor model [23], focusing on three main dimensions that are extraversion, neuroticism, and psychoticism. These classifications and the other most important systems of organization of psychopathology share the model of continuity, where the pattern of inflexibility, rigidity, and pervasiveness leads to shape personality traits into personality disorders [19, 24]. The continuum means that at one end, there are individuals showing a good psychological functioning in every domain, and at the other end, people who respond with inflexibility (in cognition, affectivity, interpersonal functioning, and impulse control levels) to the life demands. The role of personality has been increasingly shown as influencing the chronic progression of some disorders, and it has been linked to the clinical outcome of headache and MOH as well. Indeed, personality contributes to shape behavioural/life-style patterns that may trigger headache attacks [25]. Hence, the study of the associations between personality and headache has received a growing interest in the literature over time [26–28]. Investigations aimed to compare personality traits in subgroups with different headache diagnoses have highlighted that patients with chronic headache and MOH are more likely to be socially introverted compared to episodic headaches patients [29, 30]. In this direction, Silberstein and colleagues [27] found that migraine patients often have a higher level of neuroticism and vulnerability to negatively affect compared to controls. In the same direction, different studies reported that migraineurs tend to show higher scores in neuroticism than controls [19, 20, 31]. A recent meta-analysis [20] demonstrated the existence of specific personality traits in migraine, reporting, respectively, higher and lower levels of neuroticism and extraversion (evaluated according to the three-factor model) in migraineurs compared to controls. Moreover, higher levels of harm avoidance, persistence, and lower of self-directedness (evaluated according to the psychobiological model) emerged in migraineurs compared to controls.

Recent evidence reported that personality predicted the response to drug treatment, inasmuch as the early detection of personality characteristics could improve the management and outcome of CM [32, 33]. Thus, the study of personality may give new insights and ways to plan psychological interventions for headache patients and comprehend patterns driving psychiatric comorbidity.

The principal aim of the present study is to realize a systematic review focusing on the specific personality traits associated with CH compared to healthy controls (phase one). In this light, personality refers to enduring characteristics affecting an individual's behaviour and explaining consistencies across different life domains and situations. A second aim is to realize a quantitative meta-analysis with the studies that used the same instrument to assess personality (phase two).

## 2. Phase One: Systematic Review and Qualitative Meta-Analysis

### 2.1. Materials and Methods

#### 2.1.1. Search Strategy

**Figure 1:**
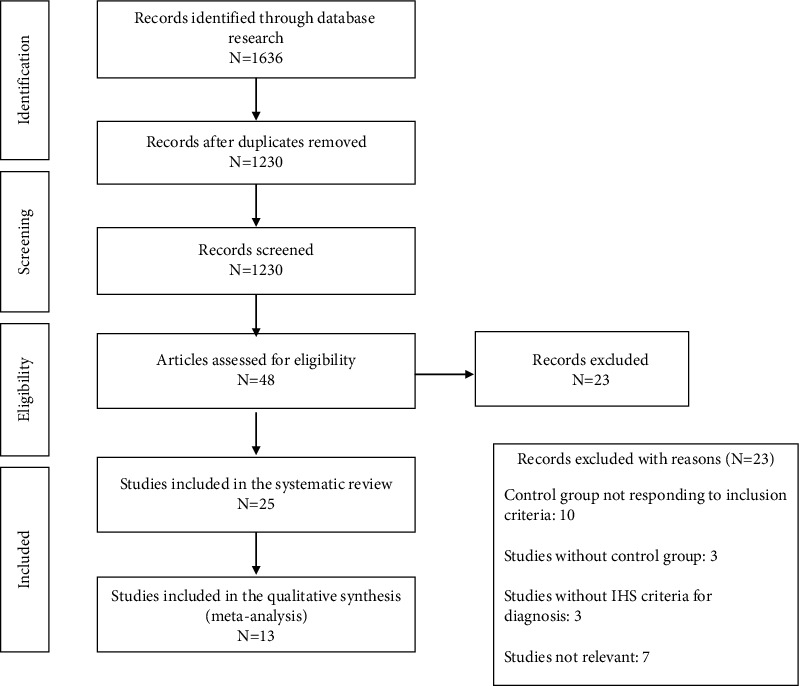
Flowchart of the selection process of the primary studies (PRISMA).

To include the broadest range of relevant literature, the electronic literature search was conducted by using major databases in the field of health and social sciences: PubMed, Scopus, PsychInfo, and Web of Science. The search was performed using the following keywords: “chronic migraine” OR “chronic headache” OR “medication overuse headache” OR “high frequency migraine” OR “chronic tension-type headache” OR “continuous headache” OR “frequent migraine” OR “refractory headache” OR “refractory migraine” OR “persistent headache” AND personality OR temperament OR “personality disorder” OR “psychometric” OR “psychogenic” OR “psychological”. The search was limited to English-language journal articles and was adapted for each database as necessary. We limited our search to the period from 1988 to the present to include only papers with International Headache Society criteria. Moreover, we performed an additional analysis of each reference list in each selected paper to ensure that all significant papers were included in the review (see Figure 1). The electronic bibliographic search was conducted in December 2022. The present review was not registered.

*(1) Selection Criteria and Data Extraction*. The following inclusion criteria were employed: (a) diagnosis of CH (MOH, CM, or CTTH) based on ICHD criteria; (b) temporal range from 1988 to the present; (c) the use of standardized and validated instruments to assess personality; (d) presence of a healthy control group; (e) written in the English language.

The exclusion criteria were as follows: (a) case reports, conference proceedings reviews, and studies reported in letters to the editor; (b) studies that enrolled children and adolescents; (c) studies that did not specify the selection criteria.

Study selection was performed by three independent reviewers who assessed the relevance of the studies' questions and objectives. This first round of selection was based on the title, abstract, and keywords of each study. Duplicate studies were removed from the list. If the reviewers did not reach a consensus or the abstracts did not contain sufficient information, the full text was reviewed. In the second phase of the study selection process—based on the papers' full text—we tested whether the studies met the inclusion criteria. Discrepancies between reviewers were resolved by a process of discussion/consensus building by a third reviewer. When the full text was not retrievable, the study was excluded.

A standardized data extraction form was prepared, and data were inserted by the three independent reviewers in a study database. The form included the following information: title, year of publication, numbers of patients and healthy controls, gender ratio, IHS criteria used to classify migraine, tools used in the psychometric assessment, study design, significant findings, and notes and/or comments on the study findings and/or design.

### 2.2. Risk of Bias

Quality assessment of each study included in the systematic review was evaluated according to the Newcastle-Ottawa Scale (NOS) for case-control studies method that is based on a 9-star model [34]. Studies scoring above the median NOS value were considered as high quality (low risk of bias), and those scoring below the median value were considered as low quality (high risk of bias) [35]. Three judges (SB, FG, and AR) independently extracted the information from all eligible reports useful to meet the above inclusion criteria.

### 2.3. Results

Figure 1 shows the flow diagram based on the PRISMA statement. The initial search identified 1636 articles. After removing duplicates, we obtained 1230 articles. According to the exclusion and inclusion criteria after full-text assessment of 41 studies, 25 were considered eligible for systematic review, 13 for a qualitative meta-analysis [19, 24, 28, 36–45], and 3 for a quantitative meta-analysis [36, 39, 40] (see Table 1).

Six studies obtained a median NOS value of 5, five studies were above it, and two were below the median value (Table 2). Thus, five studies were quoted as having high quality (low risk of bias) according to the NOS method (see Table 2).

## 3. Phase Two: Quantitative Meta-Analysis

### 3.1. Materials and Methods

#### 3.1.1. Studies Selection

Three studies assessed personality according to the MMPI. Galli et al. [40] and Sances et al. [39] assessed personality by using the MMPI on MOH patients, whereas Aguirre et al. [36] used it on CTTH patients, which were considered as reference groups and compared with healthy controls. Furthermore, Galli et al. [40] included also a group of substance addicts, which was excluded from the analysis (see Table 2).

#### 3.1.2. Statistical Analysis

Data were analysed using R 4.0.2. We computed the effect sizes (ES) from considered studies, according to means and standard deviations, using Cohen's d approach. Negative values indicated that headache had lower scores than controls in the considered outcome. Conventionally, it is considered that a value of Cohen's *d* < 0.20 indicates a small effect size (ES)=0.5, a medium ES, and >0.80, a large ES. For each effect size, we computed 95% CI, variance, standard error, and statistical significance. The random-effects model was used since it allows accounting for different sources of variation among studies in a conservative way. Statistical heterogeneity was assessed with *Q* and *I*^2^. A significant Q value represents a lack of homogeneity of findings among studies, whereas *I*^2^ allows estimating the proportion of observed variance reflecting real differences in ES. *I*^2^ is used, which usually is considered as a value of low (25%), moderate (50%), and high (75%) heterogeneity. *Q* has small statistical power in small meta-analysis, whereas *I*^2^ is independent of the number of studies. The heterogeneity among considered studies is partially tackled by the choice of the random-effects model. Through the funnel plot and Egger's performed whenever possible (with reference to the small number of studies in the analysis), the publication bias was estimated.

### 3.2. Results

#### 3.2.1. Personality Profile according to MMPI: Clinical Scale

Forest plots for MMPI clinical scales are reported in Table 3.

 
*Depression*. Both Sances et al. [39] and Aguirre et al. [36] found differences across groups, with CH patients having significantly higher scores than controls, whereas Galli et al. [40] did not. The ES (=0.826; 95% CI = [0.218–1.434]) was significant (*p*=0.0077). The heterogeneity across studies was significant (*p*=0.0006) and *I*^2^ high.
 
*Hypochondriasis*. The three studies found differences across groups, with CH patients having significantly higher scores than controls. The ES (=1.538; 95% CI = [0.742–2.335]) was significant (*p*=0.0002). The heterogeneity across studies was significant (*p* < 0.0001) and *I*^2^ high.
 
*Hysteria*. The three studies found differences across groups, with CH patients having significantly higher scores than controls. The ES (=0.992; 95% CI = [0.529–1.454]) was significant (*p* < 0.0001). The heterogeneity across studies was significant (*p*=0.014) and *I*^2^ high.
 
*Hypomania*. The three studies did not find differences in this scale across groups. The ES (=0.112; 95% CI = [−0.165–0.389]) was not significant (*p*=0.43). The heterogeneity across studies was not significant and *I*^2^ low.
 
*Paranoia*. Both Sances et al. [39] and Aguirre et al. [36] found differences across groups, with CH patients having significantly higher scores than controls, whereas Galli et al. [40] did not. The ES (=0.514; 95% CI = [0.075–0.952]) was significant (*p*=0.022). The heterogeneity across studies was significant (*p*=0.016) and *I*^2^ high.
 
*Psychopathic Deviate*. The three studies did not find differences in this scale across groups. The ES (=0.116; 95% CI = [−0.099, 0.331]) was not significant. The heterogeneity across studies was not significant and *I*^2^ low.
 
*Psychastenia*. Both Sances et al. [39] and Aguirre et al. [36] found differences across groups, with CH patients having significantly higher scores than controls, whereas Galli et al. [40] did not. The ES (=0.610; 95% CI = [0.036–1.184]) was significant (*p*=0.037). The heterogeneity across studies was significant (*p*=0.0014) and *I*^2^ high.
 
*Schizophrenia*. Both Sances et al. [39] and Aguirre et al. [36] found differences across groups, with CH patients having significantly higher scores than controls. The ES (=0.665; 95% CI = [0.012–1.317]) was significant (*p*=0.046). The heterogeneity across studies was significant (*p*=0.002) and *I*^2^ high.
 
*Social Introversion*. Only Aguirre et al. [16] found differences in this scale across groups, whereas Sances et al. [19] and Galli et al. [40] did not. The ES (=0.440; 95% CI = [−0.092–0.972]) was not significant (*p*=0.11). The heterogeneity across studies was significant (*p*=0.002) and *I*^2^ high.

## 4. Discussion

The present study focused on the investigation of personality traits that characterized patients suffering from CH compared to healthy controls. Thirteen studies were included in the systematic review, and three of them were explored in a meta-analysis, since in these studies, the same personality inventory has been employed: the MMPI. As regards the studies included in the meta-analysis [36, 39, 40], all reported that the CH patients scored higher than healthy controls in hypochondriasis and hysteria [46, 47]. Since the CH patients evaluated in the three studies suffered from MOH or CTTH, it is possible to hypothesize that these personality traits are common to different categories of CH patients. This result appears to be coherent with those of previous studies on the evaluation of personality traits in headache patients through the use of MMPI that highlighted the presence of a “neurotic profile” [46, 47]. According to this profile, headache patients are characterized by high levels of depression, hypochondria, and hysteria [29, 39, 48]. In our meta-analysis, higher levels of depression emerged only in two studies: Sances et al. [39] and Aguirre et al. [36]. However, depression, though evaluated through different tools, is highly represented in this clinical population (see Table 1). In Rausa et al. [46], the “neurotic triad” emerged only in patients with psychiatric comorbidity, while patients without psychiatric comorbidity displayed a high score only in the hypochondriasis subscale, indicating high concerns for their health status as the most central personality trait. However, the presence of the “neurotic profile” in CH [39, 40] has been considered a reaction to the chronic pain rather than a specific personality trait characterizing headache patients [49]. In this direction, hypochondriasis, characterized by a preoccupation of having a serious illness based on misrepresentation of bodily sensation persisting to reassurance [50], appeared to be commonly associated with somatization, chronic pain, and the severity of pain [50].

On the contrary, clinical and control subjects constantly did not report any difference in hypomania and psychopathic deviation. It can be hypothesized that these traits could be more relevant in people suffering from mental health problems, as they can predispose individuals to develop other disorders, instead of somatic problems such as headache. In this direction, headache patients showed a level of functioning comparable to those of healthy controls.

Moreover, only Aguirre et al. [36] found differences between clinical and control subjects as regards Social Introversion Scores. It should be noted that Aguirre et al. [36] explored personality traits in CTTH, whereas Galli et al. [40] and Sances et al. [39] in MOH. Therefore, it may be hypothesized that the social introversion could be a peculiar trait of CTTH. Specifically, tension-type forms seem to originate in emotional difficulties and stressful conditions, as may be the social circumstances; thus, individuals suffering from these forms may be more likely to avoid the situation that may provoke tension and headache thus resulting in more social introversion. Moreover, this seems to be in line with Barton–Donovan and Blanchard's [51] results that reported a higher score of social introversion in CH patients compared to less severe migraine forms.

In conclusion, the studies included in the meta-analysis [36, 39, 40] seem to define CH patients' personalities as characterized by neurotic concern over bodily functioning, hysteria, and/or physical complaints. Shyness and tendency to withdraw from social contacts and responsibilities characterize CTTH patients.

All the studies that investigated depressive and anxious personality traits constantly found higher scores in clinical groups compared to healthy controls [19, 37, 41–45]. This seems to highlight that the headache clinical population is characterized by a reduced hope in the future and general dissatisfaction with one's life, as well as the tendency to perceive things as threatening where others might not. These findings appeared to be in line with the broader literature reporting high levels of depression and anxiety in this clinical population as principal psychological comorbidities [52, 53].

A further personality trait investigated in headache patients is the pain catastrophizing [41, 42, 45] being characterized by exaggerated and negative cognitive and emotional schema brought to bear during actual or anticipated painful stimulation, the tendency to magnify or exaggerate the threat value or seriousness of pain sensations, and helplessness and ruminative thinking about pain. High levels of pain catastrophizing in headache patients and catastrophizing about consequences of somatic symptoms seem to affect headache pain intensity also [54].

As regards differences between subcategories of patients, it should be noted that MOH patients seem to be the most pathological/frail ones with scores constantly higher than migraine and TTH in hypochondriasis, health concerns, depression, hysteria, pain catastrophizing, neuroticism as the tendency to frequently experience negative emotions, and anxiety and affect regulation disorders (see Table 1). Moreover, MOH patients reported lower levels of openness, agreeableness, and consciousness that are generally considered functional personality traits (see Table 1). Such a “more complicated” characterization of MOH patients is in line with previous studies [33, 55–57] showing the causative role of psychological and psychosocial aspects in the development and perpetuation of this condition.

Consistent with previous findings, patients suffering from frequent or chronic forms of both migraines and TTH resulted characterized by higher levels of dysfunctional traits and symptoms than episodic forms [51].

The present investigation should be interpreted in the light of some limitations. First, the different instruments used for assessing personality traits and dimensions. This clinical heterogeneity made it difficult to draw firm conclusions; however, all studies included validated measures of personality; thus, a scientific criterion has been respected. A further limit is in the sample size of the studies since some of them included a limited clinical sample (*n* ≤ 65 participants) whereas others included a control group with a lower number of individuals compared to clinical subjects or vice versa. A third limit regards the gender, as unfortunately, data were not constantly available for men and women separately. Lastly, the cross-sectional nature of the studies does not allow us to draw conclusion on the direction of the association that emerged since it is not possible to conclude whether some personality traits play a role in the development of headache disorders or the prolonged pathological condition causes some changes in personality as a maladjustment to pain. Longitudinal studies are needed to draw firm conclusions on the role of personality in the evolution and outcome of headache disorders. In addition, it should be considered that although, to date, there are a large number of works devoted to the investigation of personality in headache, only a small number of these have used validated diagnostic criteria and assessment tools. Consequently, more methodological rigor would be needed in the future so as to obtain comparable data.

### 4.1. Conclusions

In conclusion, the results of this literature review with meta-analysis provide evidence supporting that MOH and CTTH are characterized by higher levels of dysfunctional personality traits and psychopathological symptoms. Insufficient evidence was available for CM. The principal personality traits involved in the onset are identified, and the maintenance of headache disorders seems to be important for the disease in order to develop specific psychological intervention programs positively influencing the health status of headache sufferers and improving their quality of life.

## Figures and Tables

**Table 1:** Characteristics of primary studies included in the meta-analysis.

Study	*N* (female : male) headache	*N* (female : male) healthy controls	Headache diagnosis	Questionnaires	Study design	Outcome	Significant findings compared to control(s)	Note
Aguirre et al., 2000	51 CTH (44 : 6)	50 (NR)	ICHD I edition	MMPI	Cross-sectional study	(i) MMPI profile (ii) Personality predictors of therapeutic response	(i) Cluster 1 (*n* = 16): significant elevations on the Hypochondria (*p*=0.0001), Depression (*p*=0.03), Paranoia (*p*=0.007), and Hysteria (*p*=0.02) Scales (ii) Cluster 2 (*n* = 33): significant elevations on hypochondria (*p*=0.0001), depression (*p*=0.0001), hysteria (*p*=0.0001), psychopathia (*p*=0.0001), paranoia (*p*=0.0001), psychosthenia (*p*=0.0001), schizophrenia (*p*=0.0001), mania (*p*=0.004) and social introversion (*p*=0.0001) (iii) No differences in therapeutic response between the two clusters	The clusters did not differ for headache frequency, intensity, or demographic variables
Cao et al., 2002	(i) 72 CTTH (36 : 36) (ii) 33 ETTH (18 : 15) (iii) 15 MA (10 : 5) (iv) 57 MO (36 : 21)	58 (40 : 18)	ICHD I edition	(i) Zuckerman-Kuhlman's personality questionnaire (ii) Plutchik-van Praag's depression inventory	Cross-sectional study	(i) Personality and depression in different headache subtypes	(i) Headache groups scored higher than healthy controls on Neuroticism-Anxiety (*p* < 0.001), Aggression-Hostility (*p* < 0.01), and PVP Depression Scores (*p* < 0.001) (ii) CTTH (*p* < 0.01 and 0.01), ETTH (*p* < 0.01 and <0.05), and MO (*p* < 0.01 and <0.05) groups scored higher on neuroticism-anxiety and PVP depression, respectively (iii) MO group (only) scored higher on aggression-hostility than controls (*p* < 0.01)	(i) Not significant differences between all headache subtypes but MA
Wang et al., 2005	41 CTTH (16 : 25) 34 FETTH (13 : 21) 48 MO (39 : 9)	37 (21 : 16)	ICHD-II edition	Dimensional assessment of personality pathology	Cross-sectional study	(i) Personality disorders in primary headaches	(i) Patients scored significantly higher than HC on submissiveness (*p* < 0.05), cognitive distortion (*p* < 0.01), identity problems (*p* < 0.05), intimacy problems (*p* < 0.01), social avoidance (*p* < 0.05), and self-harm (*p* < 0.001) (ii) Submissiveness was elevated in MIG when compared to FETTH; identity problems were lowered in FETTH but not in either CTH or migraine when compared to controls; social avoidance was lowered in CTTH and migraine, but not in FETTH when compared to controls	

Sances et al., 2010	82 MOH (62 : 20) 82 EH (63 : 19)	55 (26 : 29)	ICHD-II edition	MMPI	Cross-sectional study	(i) Personality in MOH	(i) MOH scored higher than EH in the Hypochondriasis (*p*=0.007) and Health Concerns (*p*=0.005) Scales; (ii) MOH and EH did not differ in the dependence-related behaviour scales (Addiction potential scale and Addiction Admission Scale); (iii) MOH and EH scored higher than HC in the neurotic scales (hypochondriasis, depression, hysteria) and in other scales such as Paranoia, Psychastenia, and Schizophrenia (iv) MOH and EH scored lower than HC on Ego Strength and Dominance Scales	
Galli et al., 2011	82 MOH (62 : 20)	37 (17 : 20)	ICHD-II edition	MMPI-2	Cross-sectional study	(i) Personality in MOH	MOH scored higher on hypochondriasis, depression (only females), hysteria (only females) (*p* < 0.0001)	
Radat et al., 2013	17 MOH (13 : 4) 19 EM (14 : 5)	17 (13 : 4)	ICHD-II edition	(i) BDI (ii) STAI (iii) PCS (iv) MDQ-H	Cross-sectional study	(i) Anxiety, depression, catastrophizing, and impulsivity dyscontrol in MOH (ii) Psychological correlates of prognosis in a 1-year follow-up	(i) MOH scored higher than both EM and HC in MDQ-H and PCS, differing from each other (ii) MOH and EM scored higher than HC in BDI and STAI (iii) No group differences in BIS (iv) Higher PCS scores (*p*=0.005) predicted risk of relapse	Small sample size
Kayhan, ilik 2016	105 CM (53 : 52)	100 (50 : 50)	ICHD-II edition	(i) SCID-II (ii) MIDAS	Cross-sectional study	(i) Prevalence of PDs in patients with CM	(i) 85 (81%) were diagnosed with a PD (ii) PDs were more common in the CM group than in the control group (*p* < 0.0001) (iii) Prevalence of PDs: obsessive-compulsive (50.5%), dependent (19%), avoidant (19%), and passive-aggressive (13.3%) PDs (iv) MIDAS scores of the CM patients with a PD were higher than those of CM patients without a PD (*p* < 0.0001)	
Ashina et al., 2017	(i) 83 migraine and TTH (ii) 43 pure migraine (iii) 97 pure TTH (NR)	324 (NR)	ICHD-3 beta	(i) Eysenck personality Questionnaire (ii) Major depression inventory	Cross-sectional study (general population)	(i) Relationship of neuroticism and depression with type and frequency of headache	(i) Individuals with more frequent headaches and multiple headache types have higher neuroticism and depression vs no headache and episodic headache (*p* < 0.001) (ii) Migraine: No correlation between days with headache per year and depression or neuroticism. TTH: days with headache were associated with depression but not neuroticism (*p* < 0.001)	(i) Poor description of sample characteristics (ii) Most significant findings were related to headache frequency
Mose et al., 2019	94 MOH (65 : 29) 94 migraine (82 : 12)	1032 (453 : 579)	ICHD‐III (beta)	(i) NEO‐FFI‐3 (brief version of NEO personality inventory revised)	Cross-sectional study	(i) To investigate personality characteristics by comparing the two clinical groups with a normative sample	(i) Openness, agreeableness, and conscientiousness: migraine scored higher compared to the MOH (*p* < 0.01) (ii) Neuroticism: MOH had a higher score versus controls (*p* < 0.01) (iii) Openness and agreeableness: MOH had a lower score compared to controls (*p* < 0.01) (iv) Conscientiousness: MOH group had a lower score (*p* < 0.01), whereas migraine had a higher score than controls (*p* < 0.01)	Patients with comorbid severe untreated depression, anxiety, PDs, or other pain were excluded
Consonni et al., 2020	42 CM (39 : 3)	13 (4 : 9)	ICHD-3	PCS-I HADS UCLA loneliness scale SF-12 CSQ EUROHIS-QOL-8 item COVID-19 distress questionnaire	Cross-sectional study	To evaluate the effect of COVID-19 on CM symptoms compared to controls	CM scored higher than controls on pain catastrophizing and CSQ catastrophism (*p*=0.002) CM scored significantly lower in quality of life (*p*=0.04) and physical health (*p* < 0.001)	Control group = healthy family members; Tests both in the presence/sent by e-mails
Cosci et al., 2020	100 CM (80 : 20) 100 EM (80 : 20)	100 (80 : 20)	ICHD‐III (beta)	MIDAS BPI SCID-5 SSI-DCPR-R CID ES PSI MPQ PP	Cross-sectional study	To explore whether mental pain and PP are more prevalent in CM than EM and HC	CM scored higher than HS on BPI emotional interference (*p*=0.02) and working interference (*p*=0.02), MIDAS total (*p* < 0.001) and MPQ (*p* < 0.001).CM had higher rates of major depressive episodes (*p*=0.02), allostatic overload (*p*=0.004), illness denial (*p*=0.03), and persistent somatization (*p*=0.009) and lower rates of health anxiety (*p*=0.003) and type a behavior (*p*=0.009) than controls. CM had significantly higher levels of anxiety (*p* < 0.001), depression (*p*=0.045) and lower levels of euthymia (*p* < 0.001) EM scored higher on MIDAS total (*p* < 0.001) and had higher rates of illness denial (*p*=0.014) than controls CM scored higher than EM on MIDAS total (*p* < 0.001), MPQ (*p* < 0.001) and BPI scales (all *p* < 0.03), had higher rates of persistent somatization (*p* < 0.05) and irritable mood (*p*=02), depression (*p*=0.002), psychological distress (*p* < 0.001), and abnormal illness behaviour (*p* < 0.02) than EM, as well as lower levels of euthymia (*p*=0.002)	

Migliore et al., 2020	48 MOH (38 : 10)	48 HC (37 : 11)	ICHD-3 beta	BDI-2 STAY-Y DERS TAS-20 BIS-11	Case-control study	Psychopathological profiles in MOH patients	MOH scored significantly higher than HC on DERS total and subscales (all *p* < 0.01; except for goal subscale *p*=*ns*), TAS-20 total (*p*=003) and DIF (*p* < 0.001), BIS-11 attention scale (*p*=0.006) BDI-2 (*p* < 0.001) and STAY-Y (*p* < 0.001)	Subjects reporting medical conditions and neurological or psychiatric diseases were excluded
Pistoia et al., 2022	65 CM (65 : 0) 65 EM (65 : 0)	65 HC (65 : 0)	ICHD-3	PSQI ISI ESS STAI-X2 ASI-3 BDI-II IUI-10 IUS-12 URS IA PCS-I GDMS	Cross-sectional study	To investigate specific behavioural and psychological factors in migraine To identify a specific mindset associated with migraine	CM showed greater trait anxiety (*p* < 0.001) and reported higher pain catastrophizing tendency, feeling of helplessness, and ruminative thinking than HC (all *p* < 0.001) EM reported more severe pain catastrophizing tendency, feeling of helplessness, and ruminative thinking compared to HC (*p*=0.013; *p*=0.007; *p*=0.009; respectively) CM reported higher sensitivity to anxiety symptoms (*p*=0.047), pain catastrophizing tendency, feeling of helplessness, and ruminative thinking compared to the EM group (*p*=0.003; *p*=0.002; *p*=0.007; respectively)	Only female participants Patients with a history of psychiatric comorbidities were excluded

NR: not reported; M: migraine; CM: chronic migraine; TTH: tension-type headache; EH: episodic headache; EM: episodic migraine; FETTH; frequent episodic tension-type headache; SA: substance addiction; MOH: medication-overuse headache; CTTH: chronic tension-type headache; ETTH: episodic tension-type headache; TCI: temperament and character inventory; BD: blood donors; HGHP: historical group with healthy people; NMCP: no migraine chronic pain; PD: personality disorder; PSE-10: present state examination; BDI: beck depression inventory; SCID-I: structured clinical interview for DSM-IV axis I disorders; SCID-II: structured clinical interview for DSM, personality disorders; STAXI: state-trait anger expression inventory; MIDAS: migraine disability assessment score questionnaire; MMPI-2: Minnesota Multiphasic Personality Inventory-2; ICHD: international classification of headache disorders; DSM-III R: diagnostic and statistical manual of mental disorders, third edition; BPI: brief pain inventory; SSI-DCPR-R is a semistructured interview based on the diagnostic criteria for psychosomatic research–revised; CID: clinical interview for depression; ES: Euthymia Scale; PSI: psycho-social index; MPQ: mental pain questionnaire; PP: pain-proness checklist; HADS: Hospital Anxiety and Depression Scale; SF-12 : 12-item short-form survey; PCS-I: Pain Catastrophizing Scale-I; CSQ: coping strategies questionnaire; EUROHIS-QOL 8-item: EUROHIS-quality of life 8-item index; DERS: Difficulties in Emotion Regulation Scale; TAS-20: Toronto Alexithymia Scale-20 item; BIS: Barratt Impulsiveness Scale; BDI-2: beck depression inventory-2; STAI-Y, state-trait anxiety inventory-Y; PSQI: pittsburgh sleep quality index; ISI: insomnia severity index; ESS: Epworth Sleepiness Scale; STAI-X2: state-trait anxiety inventor-X2; ASI-3: anxiety sensitivity index-3; IUI-10 intolerance of uncertainty inventory-10 item, IUS-12: Intolerance of Uncertainty Scale-12 item; URS: Uncertainty Response Scale; IA: intolerance of ambiguity questionnaire; GDMS: general decision-making style.

**Table 2:** Risk of bias of the included studies.

Authors	Selection				Comparability	Outcome			NOS total
	Adequate case definition	Representativeness	Selection of controls	Definition of controls		Ascertainment	Same ascertainment for case/control	Nonresponse rate	
Aguirre et al., 2000	^*∗*^	^*∗*^	^*∗*^	^*∗*^	—	—	^*∗*^	*N*	5
Cao et al., 2002	^*∗*^	—	^*∗*^	^*∗*^	—	*N*	^*∗*^	*N*	4
Wang et al., 2005	^*∗*^	—	^*∗*^	^*∗*^	—	—	^*∗*^	*N*	4
Sances et al., 2009	^*∗*^	^*∗*^	^*∗*^	^*∗*^	—	—	^*∗*^	*N*	5
Galli et al., 2011	^*∗*^	^*∗*^	^*∗*^	^*∗*^	—	—	^*∗*^	*N*	5
Radat et al., 2013	^*∗*^	^*∗*^	^*∗*^	^*∗*^	—	*N*	^*∗*^	*N*	5
Kayhan, ilik, 2016	^*∗*^	^*∗*^	^*∗*^	^*∗*^	—	^*∗*^	^*∗*^	*N*	6
Ashina et al., 2017	^*∗*^	—	*N*	^*∗*^	^*∗∗*^	*N*	^*∗*^	^*∗*^	6
Mose et al., 2019	^*∗*^	^*∗*^	^*∗*^	^*∗*^	—	—	^*∗*^	*N*	5
Consonni et al., 2020	^*∗*^	^*∗*^	^*∗*^	^*∗*^	—	—	^*∗*^	*N*	5
Cosci et al., 2020	^*∗*^	^*∗*^	^*∗*^	^*∗*^	^*∗*^	—	^*∗*^	^*∗*^	7
Migliore et al., 2020	^*∗*^	^*∗*^	^*∗*^	^*∗*^	^*∗*^	—	^*∗*^	—	6
Pistoia et al., 2022	^*∗*^	^*∗*^	^*∗*^	^*∗*^	^*∗*^	—	—	^*∗*^	6

^*∗*^The criterion is reflected in the study. ^*∗∗*^Two stars were assigned when the control was matched not only for age and gender.

**Table 3:** Forest plot for clinical scales of the Minnesota Multiphasic Personality Inventory (MMPI) for chronic headache patients (case) and controls.

Study	Case		Control		Weight (%)	Effect size	Effect size
	*N*	*M* (SD)	*N*	*M* (SD)		M-H, random, 95% CI	M-H, random, 95% CI
Depression							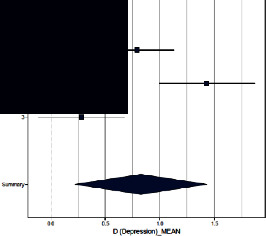
(1) Sances et al., 2009	82	26.4 (7.0)	55	21.5 (4.9)	34.5	0.790 [0.451–1.130]	
(2) Aguirre et al., 2000	51	64.5 (9.2)	50	52.0 (8.3)	32.2	1.430 [0.992–1.868]	
(3) Galli et al., 2011	82	25.3 (16.3)	37	21.0 (11.7)	48.1	0.280 [−0.112–0.672]	
Summary						0.826 [0.219–1.434]	
Heterogeneity: Tau^2^ = 0.249; *Q* = 14.69, df = 2 (*p*=0.0006); *I*^2^ = 86.4%							
Test for overall effect: *Z* = 2.66 (*p*=0.007)							

Hypochondriasis							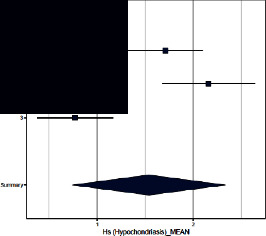
(1) Sances et al., 2009	82	14.2 (5.5)	55	5.9 (3.6)	33.8	1.710 [1.318–2.102]	
(2) Aguirre et al., 2000	51	69.0 (9.7)	50	49.6 (8.2)	32.4	2.160 [1.680–2.640]	
(3) Galli et al., 2011	82	12.8 (12.4)	37	4.5 (5.3)	33.8	0.770 [0.370–1.162]	
Summary						1.539 [0.742–2.335]	
Heterogeneity: Tau^2^ = 0.448; *Q* = 21.63, df = 2 (*p* < 0.0001); *I*^2^ = 90.8%							
Test for overall effect: *Z* = 3.79 (*p*=0.0002)							

Hysteria							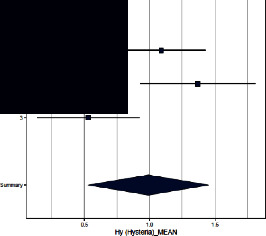
(1) Sances et al., 2009	82	27.3 (5.5)	55	21.9 (3.9)	35.4	1.090 [1.751–1.430]	
(2) Aguirre et al., 2000	51	63.0 (8.6)	50	50.9 (9.1)	31.4	1.370 [0.932–1.808]	
(3) Galli et al., 2011	82	26.75 (9.06)	37	22 (9.01)	33.3	0.530 [0.138–0.922]	
Summary						0.992 [0.529–1.454]	
Heterogeneity: Tau^2^ = 0.127; *Q* = 8.51, df = 2 (*p*=0.014); *I*^2^ = 76.5%							
Test for overall effect: *Z* = 4.20 (*p* < 0.0001)							

Hypomania							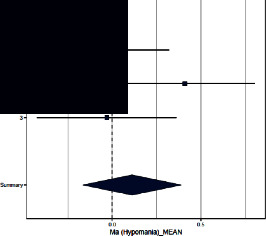
(1) Sances et al., 2009	82	15.0 (4.4)	55	15.0 (4.9)	37.2	1.090 [1.751–1.430]	
(2) Aguirre et al., 2000	51	47.5 (9.3)	50	43.9 (8.3)	31.4	1.430 [0.992–1.868]	
(3) Galli et al., 2011	82	13.8 (8.5)	37	14.0 (10.4)	31.4	0.530 [0.138–0.922]	
Summary						0.992 [0.529–1.454]	
Heterogeneity: Tau^2^ = 0.024; *Q* = 3.30, df = 2 (*p*=0.20); *I*^2^ = 39.4%							
Test for overall effect: *Z* = −0.79 (*p*=0.43)							

Paranoia							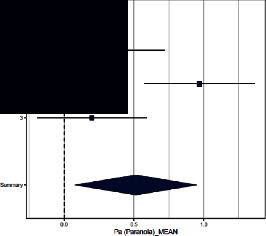
(1) Sances et al., 2009	82	10.3 (4.8)	55	8.8 (2.8)	34.8	0.380 [0.041–0.720]	
(2) Aguirre et al., 2000	51	55.8 (10.2)	50	47.0 (7.7)	32.6	0.970 [0.578–1.362]	
(3) Galli et al., 2011	82	9.5 (8.5)	37	8.0 (4.5)	32.6	0.200 [−0.192–0.592]	
Summary						0.514 [0.075–0.952]	
Heterogeneity: Tau^2^ = 0.114; *Q* = 8.25, df = 2 (*p*=0.016); *I*^2^ = 75.8%							
Test for overall effect: *Z* = 2.30 (*p*=0.022)							

Psychopathic deviate							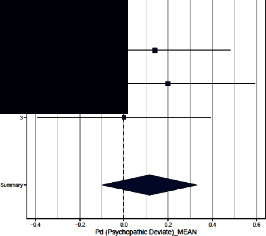
(1) Sances et al., 2009	82	17.6 (6.7)	55	16.8 (4.4)	40.0	1.140 [−0.200–0.480]	
(2) Aguirre et al., 2000	51	53.3 (11.2)	50	51.4 (7.6)	30.0	0.200 [−0.192–0.592]	
(3) Galli et al., 2011	82	15.0 (11.3)	37	15.0 (6.0)	30.0	0.000 [−0.392–0.392]	
Summary						0.116 [−0.099–0.331]	
Heterogeneity: Tau^2^ = 0; *Q* = 0.53, df = 2 (*p*=0.77); *I*^2^ = 0.0%							
Test for overall effect: *Z* = 1.06 (*p*=0.289)							

Psychastenia							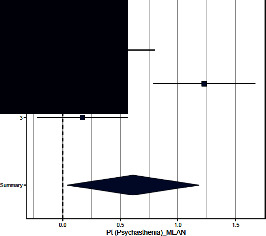
(1) Sances et al., 2009	82	16.4 (9.8)	55	12.4 (7.2)	34.6	0.460 [0.121–0.800]	
(2) Aguirre et al., 2000	51	59.4 (10.5)	50	48.5 (6.7)	32.1	1.230 [0.792–1.669]	
(3) Galli et al., 2011	82	13.0 (24.1)	37	9.3 (13.9)	33.3	0.170 [−0.222–0.562]	
Summary						0.610 [0.036–1.184]	
Heterogeneity: Tau^2^ = 0.218; *Q* = 13.11, df = 2 (*p*=0.0014); *I*^2^ = 84.7%							
Test for overall effect: *Z* = 2.08 (*p*=0.037)							

Schizophrenia							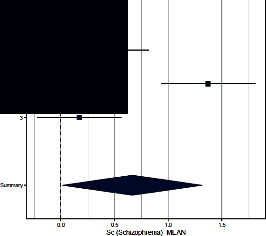
(1) Sances et al., 2009	82	15.6 (10.1)	55	11.4 (6.1)	34.3	0.480 [0.141–0.820]	
(2) Aguirre et al., 2000	51	58.4 (10.8)	50	46.6 (5.5)	32.3	1.370 [0.932–1.808]	
(3) Galli et al., 2011	82	12.8 (21.0)	37	9.8 (9.2)	33.3	0.170 [−0.220–0.562]	
Summary						0.665 [0.012–1.317]	
Heterogeneity: Tau^2^ = 0.293; *Q* = 16.96, df = 2 (*p*=0.0002); *I*^2^ = 88.2%							
Test for overall effect: *Z* = 2.00 (*p*=0.046)							

Social introversion							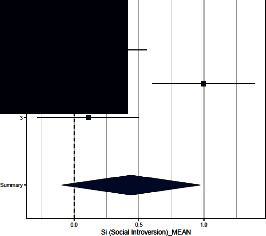
(1) Sances et al., 2009	82	31.3 (19.8)	55	29.2 (8.8)	34.4	0.220 [−0.120–0.560]	
(2) Aguirre et al., 2000	51	58.2 (9.2)	50	49.2 (8.8)	32.8	1.000 [0.608–1.392]	
(3) Galli et al., 2011	82	31.8 (20.0)	37	29.5 (21.0)	32.8	0.110 [−0.282–0.502]	
Summary						0.440 [−0.092–0.972]	
Heterogeneity: Tau^2^ = 0.185; *Q* = 12.15, df = 2 (*p*=0.002); *I*^2^ = 83.5%							
Test for overall effect: *Z* = 12.15 (*p*=0.002)							

## Data Availability

The excel data used to support the findings of this study have been deposited in the ZENODO repository (10.5281/zenodo.7714022).

## Abbreviations

*M*: Migraine
CM: Chronic migraine
TTH: Tension-type headache
EH: Episodic headache
EM: Episodic migraine
FETTH: Frequent episodic tension-type headache
MOH: Medication-overuse headache
CTTH: Chronic tension-type headache
ETTH: Episodic tension-type headache.
